# Establishing the transdiagnostic contextual pathways of emotional outbursts

**DOI:** 10.1038/s41598-022-11474-4

**Published:** 2022-05-06

**Authors:** Justin Cheuk Yin Chung, Carmel Mevorach, Kate Anne Woodcock

**Affiliations:** 1 School of Psychology, University of Birmingham, Birmingham, B15 2SA UK; 2 Centre for Applied Psychology and the Institute for Mental Health, School of Psychology, University of Birmingham, Birmingham, B15 2SA UK

**Keywords:** Emotion, Human behaviour, Developmental disorders

## Abstract

Emotional outbursts or temper outbursts are challenging behaviours commonly experienced by people with neurodevelopmental disorders and people who have experienced childhood adversity, which can negatively impact individuals and their families. Emotional outbursts may manifest in different situations via unique pathways distinguished by context-specific differences in the regulation and expression of emotions. Caregivers (*N* = 268) of young people (6–25 years) with emotional outbursts completed a bespoke caregiver-report questionnaire. Potential pathways were identified by examining the patterns of antecedents and setting events related to outbursts through factor and cluster analyses. Six contextual factors were derived from the Emotional Outburst Questionnaire. Based on these factors, the responses were classified into three clusters, which may represent potential pathways of emotional outbursts. The three clusters were characterized by the increased likelihood of outbursts: (1) across all setting events and triggers; (2) in safe setting events; (3) in unsafe setting events. These potential pathways may be related to: (1) differences in sensory processing; (2) masking of emotions in unsafe environments; (3) differences in safety perception. This framework supports a transdiagnostic account of emotional outbursts and may facilitate the development of pathway-specific intervention strategies.

## Introduction

Emotional outbursts are prevalent and developmentally persistent in people with neurodevelopmental disorders, and in people who have experienced childhood adversity or trauma (e.g., refs.^1,2^). We define an emotional outburst as a highly emotional, explosive episode, characterized by a pattern of challenging behaviour that varies across individuals and across time, but can be immediately identified by caregivers^3^. Emotional outbursts are often referred to as “temper outbursts” or “tantrums”, but other synonymous terms include “meltdowns” and “rages”^4–6^.

Phenomenological studies of emotional outbursts have been conducted in individuals with specific diagnoses: in children and adults with Prader-Willi syndrome^7,8^, Lowe syndrome^9^, and in autistic toddlers^10^. These studies revealed that the behavioural topographies of outbursts are similar across disorders, and comparable to behaviours displayed in tantrums of typically developing toddlers^3,8–11^. Consideration of the antecedents and environmental factors that mediate the likelihood of the occurrence of emotional outbursts (setting events) may be critical to the understanding of outbursts, as an emotional outburst can be considered a product of environmental and biological factors^12^. Previous studies have found that the antecedents and setting events of outbursts varied across individuals within each neurodevelopmental condition, but the overall range of these contexts between conditions appeared to be comparable^7–10^. These findings challenge the conventional perspective of considering emotional outbursts and other challenging behaviours within the bounds of diagnostic constructs, which assumes that emotional and behavioural processes are inherently distinct across individuals with different diagnoses, and that these disparities in emotion and behaviour can be adequately and solely explained by biological differences linked to the corresponding diagnoses^13^. For example, previous studies have identified sensory stimuli as causes of outbursts for some individuals^5,7,14,15^, which could be related to underlying sensory processing difficulties shared by individuals across a wide range of diagnoses (e.g., refs.^16–21^). Thus, it would be unlikely for outbursts caused by sensory stimuli to manifest in ways which are entirely specific to diagnosis.

Further compounding the issue over the utility of a diagnostic framework for studying emotion and behaviour, the diagnostic validity of two of the most common neurodevelopmental psychiatric diagnoses, autism spectrum disorder and attention deficit hyperactivity disorder (ADHD), has been put into question, given that no psychological or neurobiological marker can be consistently found across individuals with either diagnosis^22,23^. The absence of such markers in autistic individuals or individuals with ADHD reinforces the view that diagnostic status may lack explanatory power when considering the aetiology of challenging behaviours such as emotional outbursts, an endeavour which is critical in developing effective intervention. The argument against the use of diagnostic boundaries can be extended to interventions for emotional outbursts and other challenging behaviours, as common strategies, such as psychoactive medications or psychological therapies have transdiagnostic mechanisms of action at a system-level, which lack diagnostic specificity^24,25^. Indeed, policy regarding the management of challenging behaviour for people with intellectual disabilities from the United Kingdom’s National Institute for Health and Care Excellence^26^ does not place emphasis on diagnostic boundaries. In the recommendations, there is an absence of differential guidance based on the aetiology of the intellectual disability and these recommendations appear to be based on evidence involving individuals with a range of different conditions^26^.

When considering the aetiology of emotional outbursts specifically, this phenomenon has classically been regarded under a purely operant reinforcement framework^27,28^. However, this account appears to have inadequate explanatory power for emotional outbursts^29^, as anecdotal and empirical reports from caregivers and autistic young people suggest that outbursts occur due to the individuals losing control^5,14^, and that individuals frequently display remorse immediately after outbursts^8,9^. Across diverse fields, emotional outbursts have been traditionally accepted as behavioural manifestations of emotion dysregulation, as reflected by the inclusion of items regarding the existence or frequency of outbursts on emotion dysregulation subscales of both widely used (e.g., Behavior Rating Inventory of Executive Function^30^) and recently developed measures of behaviour problems (e.g., Emotion Dysregulation Inventory^31^). This has created a widely assumed one-to-one mapping between the two constructs, which empirical research to date has seldom addressed or explored in further detail. At present, the link between emotional outbursts and emotion dysregulation requires further refinement to determine how emotion dysregulation may have adequate explanatory power as an aetiological mechanism to explain the variation observed in emotional outbursts across individuals and across time^32^.

If emotion dysregulation were indeed central to the aetiology of emotional outbursts, then one might expect differences in the emotional processes that underlie the observed dysregulation (i.e., the regulation and subsequent expression of emotions) to directly influence the variability in the antecedents and setting events associated with emotional outbursts. This expectation would be consistent with the transdiagnostic perspective regarding outbursts, as emotion regulation has been proposed to be a transdiagnostic domain within the Research Domain Criteria framework, which could account for psychopathology across diagnoses^33,34^. Furthermore, emotion regulation may reciprocally interact with other domains (e.g., cognitive systems^35^), such that differences in these domains could ultimately lead to differences in emotional processes. The context-dependence of emotional processes is a source of variability that could conceivably account for the range of antecedents and setting events associated with emotional outbursts, which would enable the aetiological account of outbursts to be expanded in terms of the differences in emotion regulation or expression that might lead to dysregulation and subsequent outbursts in specific contexts^35–38^.

In this expanded framework, it is possible that an individual may experience outbursts in a given set of contexts due to a pattern of context-specific differences in emotion regulation or expression. Such a relationship between the contexts associated with outbursts and the underlying differences in emotion regulation or expression represents a distinct contextual pathway of emotional outbursts. One such pathway has been delineated in individuals with Prader-Willi syndrome who experienced outbursts in response to change-related antecedents (e.g., changes to routines). In this pathway, an impairment in the cognitive ability of task-switching was demonstrated to increase the likelihood of outbursts in response to the demands of change, and this was proposed to be mediated via the emotional impact of the interaction between the cognitive deficit and the specific environmental demand^39–41^. The present framework suggests that this pathway may be transdiagnostic, which is supported by neither change-related outbursts nor differences in task-switching being exclusive to people with Prader-Willi syndrome (e.g., refs.^9,42^). Notably however, even in people with Prader-Willi syndrome, this pathway can account for outbursts in a proportion of individuals^7^. Thus, we expect additional pathways involving other differences in emotion regulation or expression to account for discrete sets of contexts in which outbursts can occur. Furthermore, whilst biological factors, such as certain genetic syndromes, may predispose an individual to experience outbursts (e.g., ref.^43^), we hypothesise that individual differences in emotion regulation or expression ultimately determine the pathway through which emotional outbursts manifest. We further expect that these differences in emotion regulation or expression can similarly account for emotional outbursts in people who have experienced childhood adversity or trauma, as a broad range of emotional and cognitive differences linked to psychopathology have been reported in this population of individuals (for reviews, see refs.^44–46^).

In this study, informant-report questionnaire responses were used to investigate the contexts of emotional outbursts transdiagnostically using cluster analysis, with the aim of establishing some of the potential contextual pathways of outbursts. Given the scarcity of current literature around the contexts and associated mechanisms of emotional outbursts, we did not hold specific hypotheses about the nature of the pathways that would be identified. However, the analytic strategy in the present study allowed for a balance between statistical robustness and clinical interpretability to ensure meaningful results to serve as a foundation for future work in this area.

## Methods

### Participants

Participants were recruited from local, regional, and national support groups for individuals with neurodevelopmental disorders based in the United Kingdom, and national support groups in Ireland, North America, and Australia. Several local, regional, and national organizations supporting adoptive and foster families in the United Kingdom assisted in recruitment. Organisations supported recruitment of caregivers by distributing information about the study and the study survey link. The inclusion criteria were that participants must be caring for young people between the ages of 6–25 years, who experienced emotional outbursts at least once a month.

Six responses were excluded due to missing demographic information regarding age, gender, and diagnoses. Nine responses were excluded as the young person’s age did not meet the age criterion. The analysis was based on 268 responses with complete Emotional Outburst Questionnaire data. Five of these responses had partially missing demographic information. The mean age of the young people was 13.5 years (*SD* = 5.2; range = 6.1–25.9). There were 162 males (60.4%), 105 females (39.2%), and 1 non-binary individual (0.4%). The mean Social Communication Questionnaire score was 19.5 (*SD* = 8.5; range = 2–36; 1 response missing). Supplementary Table S1 presents diagnostic information of the young people.

Seventy young people were medicated for outbursts (26.2%; 1 missing). In terms of support, 130 families have accessed some form of program, training, or intervention for outbursts (49.2%; 4 missing). Of these families, 65 rated the support as effective (50.4%; 1 missing). Young people from 202 families were reported to have special education needs and disabilities (SEND; 76.5%; 4 missing). Of these families, 160 had a formal statement or plan in place for the young person’s SEND (79.2%). Regarding current schooling or employment status: 146 attended mainstream schools (54.9%); 68 attended special schools (25.6%); 11 were in further education (4.1%); 2 were in higher education (0.8%); 9 were employed or in employment preparation (3.4%); and 30 were unemployed (11.3%; 2 missing). A question regarding early traumatic and adverse experiences was added to the survey partway through data collection. In this question, traumatic or adverse events were defined as single or prolonged events causing severe stress, which are different from events typically expected to occur during childhood or adolescence. The following examples of traumatic events were provided: natural disasters; death or serious injury of someone close to the person; poverty; witnessing abuse or violence; emotional, physical, or sexual abuse; neglect. Out of 151 available responses, 73 young people were reported to have experienced early traumatic or adverse events (48.3%; 4 selected *Prefer not to say*; 117 missing). Families were based in the United Kingdom (*n* = 199; 74.3%), North America (*n* = 49; 18.3%), Australia (*n* = 10; 3.7%), and other countries (*n* = 5; 1.9%; 5 missing).

### Measures

#### Emotional outburst questionnaire

The Emotional Outburst Questionnaire consists of 133 items divided into three sections (see Supplementary Information for the full questionnaire and Supplementary Methods for details on the development of the measure). Sections 1 and 2 explore the characteristics of the most and least severe outbursts, respectively, which include the behavioural composition, frequency, duration, intensity, and recovery duration of outbursts. Section 3 queries general characteristics of outbursts, which include setting events and antecedents related to outbursts, behaviours that occur after outbursts, and caregiver management strategies effective in stopping outbursts. Items related to the setting events and antecedents of outbursts are rated on a three-point frequency rating scale with subjective and objective quantifiers: “Not applicable/never/rarely (0–3 times out of 10)”, “Sometimes (4–6 times out of 10)”, “Often/always (7–10 times out of 10)”. Informants are asked to recall outbursts that have occurred within the past month.

#### Social communication questionnaire

The Social Communication Questionnaire^47^ (SCQ) is a 40-item informant-based autism spectrum disorder (ASD) screening measure, divided into three domains: reciprocal social interaction, communication, and stereotyped patterns of behaviour. The items within the SCQ were derived from the Autism Diagnostic Interview-Revised^48^ (ADI-R). The SCQ demonstrated good internal consistency across developmental ability and age, and good convergent validity with the ADI-R^47^.

As many neurodevelopmental disorders are associated with the co-occurrence of ASD^49^, the SCQ was used to provide a common measure of social communication deficits within the sample, which may have particular relevance to the aetiology of emotional outbursts. The SCQ was selected over other similar measures, as it is an informant-report measure that is appropriate for individuals of all intellectual abilities and age, which aligned with the study inclusion criteria.

### Procedure

The study received ethical approval from the Science, Technology, Engineering and Mathematics Ethical Review Committee at the University of Birmingham and the study was conducted in accordance with relevant guidelines and regulations. After providing informed consent, caregivers completed an anonymous survey consisting of the Emotional Outburst Questionnaire, SCQ, and a demographic questionnaire. Nearly all participants completed the survey online on Qualtrics. One caregiver completed the survey on paper. The median survey completion time was 28 min (interquartile range = 43 – 22 = 21 min). The Emotional Outburst Questionnaire and demographic questionnaire were completed a second time by original participants or secondary caregivers at least 14 days after initial survey completion on a voluntary basis. Out of the 199 participants invited to complete the survey for a second time, 48 original participants and 10 secondary caregivers completed this second survey.

### Statistical analyses

As this article focuses on the contextual pathways of outbursts, analyses of items related to setting events and antecedents are presented. Statistical analysis was undertaken in R 4.0.2.

Test–retest and interrater reliability were measured in terms of Cohen’s κ with quadratic weightings.

The latent structure of the 55 items pertaining to both the antecedents and setting events of outbursts were first identified using exploratory factor analysis with maximum likelihood extraction and oblimin rotation, in order to overcome the obstacle presented by the large number of items, such that contexts with similar characteristics would be grouped into salient factors. The response options were coded as 0 = “Not applicable/never/rarely (0–3 times out of 10)”, 0.5 = “Sometimes (4–6 times out of 10)”, 1 = “Often/always (7–10 times out of 10)”. To further validate the contextual items of the questionnaire, the internal consistency of each factor was evaluated by calculating Cronbach’s α using items with loadings ≥ 0.40. Refined and non-refined factor scores were generated for subsequent analysis^50^. Refined factor scores corresponded to standardized regression-based factor scores, which accounted for all item loadings and intercorrelations amongst items and factors^50^. In contrast to refined factor scores, which accounted for all items included in the analysis, non-refined factor scores constituted unweighted averages of only items with loadings ≥ 0.40.

To identify common patterns of contexts in which outbursts occurred, the factor scores of responses were classified into clusters based on squared Euclidean distances between responses. Ward’s hierarchical agglomerative clustering^51^ was used to identify the suitable number of clusters for subsequent analysis. This approach allowed for exploration of the data at different clustering steps to determine the most appropriate cluster structure in terms of cluster interpretability. Furthermore, *k*-means clustering was performed to provide additional support for the chosen cluster structure, and to demarcate the centroid of each cluster in terms of mean factor scores. The level of agreement between cluster structures was evaluated using Cohen’s unweighted κ, which accounts for classification agreements due to chance^52^. To test for the potential impact of using factor scores on the cluster structure, hierarchical clustering was performed on the responses of the 55 items and compared to the results of hierarchical clustering using refined factor scores. There was a broad level of agreement, as cluster membership was maintained for 190 participants (70.9%; Cohen’s κ = 0.56, 95% CI [0.48, 0.64]).

Refined factor scores were the focus of the cluster analysis as these scores retained more information regarding the factor structure compared to non-refined scores. Cluster analysis was additionally conducted on non-refined factor scores to characterize cluster centroids in terms of factor scores that were less sample-dependent and easier to calculate, thus enabling cluster classification in subsequent samples.

Factor scores were compared between clusters using multivariate analysis of variance (MANOVA). As the assumption of homogeneity of variance between groups was violated for some factors, Welch’s ANOVA and post-hoc pairwise Games-Howell tests were selected as follow-up analyses based on their robustness against violations of homogeneous variances. Cluster differences were further assessed with Welch’s ANOVA and χ^2^ tests of association in terms of SCQ scores and demographic variables for which sufficient data were available. Significant ANOVA and χ^2^ tests were followed with post-hoc pairwise Games-Howell and χ^2^ tests, respectively. Effect sizes are presented as ω^2^ and Cramer’s V.

## Results

### Contextual factors

The test–retest reliability and interrater reliability of the contextual items were κ = 0.63 (95% confidence interval (CI) = 0.59, 0.68) and κ = 0.63 (95% CI = 0.54, 0.72), which indicated moderate agreement.

For the exploratory factor analysis of the contextual items, parallel analysis^53^, the Very Simple Structure criterion^54^, and Kaiser’s criterion of retaining factors with eigenvalues ≥ 1^55^ all indicated that six factors were optimal. The six-factor solution accounted for 32.4% of variance (Table 1).

**Table 1:** Loadings of contextual items from the Emotional Outburst Questionnaire onto six factors. Loadings ≥ 0.40 in bold. Loadings rounded up to 0.40 are not in bold and were not included in Cronbach’s α or non-refined factor score calculations.

Item	Factor loading					
	1	2	3	4	5	6
Separation from caregiver	**0.41**	0.07	0.14	0.21	− 0.05	− 0.03
Not understand what is going on	**0.44**	0.24	0.16	0.03	0.06	− 0.05
Light is too bright	**0.57**	− 0.09	0.12	0.10	− 0.01	0.02
Sudden or loud noises	**0.59**	0.07	− 0.08	0.09	− 0.03	− 0.07
Temperature is too hot or too cold	**0.53**	− 0.04	0.17	0.08	− 0.04	0.15
Particular smells or strong smells	**0.64**	− 0.08	0.23	− 0.03	− 0.09	0.03
Touch-related over-sensitivity	**0.58**	0.00	0.09	0.14	0.10	− 0.04
Other sensory-related triggers	**0.56**	0.10	− 0.14	0.11	0.08	0.03
Change in own routine	0.32	**0.50**	− 0.21	− 0.04	− 0.04	0.14
Change in another's routine	0.30	**0.51**	− 0.12	0.06	0.01	0.02
Change in expectation	0.07	**0.50**	0.11	− 0.07	0.02	0.06
Being fixated on a thought or idea	− 0.01	**0.45**	0.07	− 0.03	0.07	0.00
Individual’s demand not met	− 0.22	**0.58**	0.09	0.02	0.02	0.13
Individual waiting for demand to be met	− 0.10	**0.68**	− 0.03	0.08	0.00	0.05
Demand placed on individual	− 0.08	**0.62**	0.13	− 0.02	− 0.02	− 0.02
Boring task	− 0.06	**0.47**	0.21	0.07	0.09	− 0.15
Disagreement with others	− 0.06	0.10	**0.69**	0.02	0.07	0.05
Being criticized	0.01	0.08	**0.71**	− 0.04	0.10	− 0.01
Being teased	0.13	0.01	**0.70**	0.03	− 0.15	0.01
Feeling of being treated unfairly	0.06	− 0.03	**0.76**	− 0.09	0.03	0.11
Receiving conflicting information	0.35	0.15	**0.43**	0.03	0.11	− 0.12
Unsafe setting	0.00	0.16	0.04	**0.44**	− 0.32	0.11
Familiar setting	0.05	− 0.13	− 0.06	**0.45**	0.39	0.12
Public setting	0.08	0.09	− 0.25	**0.65**	0.00	0.11
Unsafe person	0.18	0.01	0.01	**0.54**	− 0.22	− 0.02
Familiar person	− 0.11	− 0.06	0.11	**0.58**	0.02	− 0.02
Unfamiliar person	0.16	0.04	− 0.10	**0.65**	− 0.02	− 0.04
A person the individual dislikes	− 0.07	0.08	0.18	**0.59**	− 0.24	0.00
Safe setting	0.03	0.05	− 0.01	− 0.11	**0.79**	− 0.01
Private setting	0.13	0.05	0.10	− 0.02	**0.51**	0.01
Safe person	− 0.06	0.03	0.03	− 0.04	**0.72**	0.08
A person the individual likes	− 0.03	− 0.19	0.07	0.30	**0.42**	0.01
Tired	− 0.08	0.08	0.04	0.05	0.13	**0.60**
Hungry or thirsty	− 0.04	− 0.01	0.15	0.04	0.05	**0.70**
Unfamiliar setting	0.14	0.07	0.01	0.40	− 0.03	0.22
A person the individual is jealous of	− 0.12	0.08	0.27	0.22	− 0.03	0.23
Consuming too much of one type of food or drink	0.05	0.05	0.09	0.04	0.01	0.34
Illness	0.27	− 0.01	− 0.26	− 0.06	− 0.18	0.40
In pain	0.38	− 0.01	− 0.18	0.01	− 0.06	0.38
In a bad mood	0.00	0.15	0.19	− 0.05	− 0.03	0.29
Planned transition	0.19	0.37	− 0.12	0.03	0.03	0.02
Specific phobia or fear	0.18	0.22	0.21	0.16	− 0.04	0.06
Food-related triggers	0.21	0.03	0.16	− 0.03	0.09	0.26
Concerns for own property	0.24	0.15	0.31	− 0.07	0.00	0.12
Difficult task	0.10	0.31	0.25	0.15	0.07	− 0.08
Repetitive task	0.03	0.40	0.09	0.13	0.14	− 0.12
New task	0.24	0.40	0.16	0.10	0.01	− 0.07
Under time pressure	0.32	0.22	0.31	− 0.09	0.09	0.04
Not receiving enough attention	− 0.14	0.35	0.15	0.06	− 0.04	0.09
Receiving too much attention	0.24	0.11	0.15	0.17	0.09	− 0.13
The Individual not being understood	0.27	0.16	0.30	0.18	− 0.11	− 0.08
Not understanding someone else	0.25	0.17	0.40	0.11	0.06	− 0.03
Medication side-effect	0.16	0.05	− 0.13	0.11	0.06	0.04
Mood of caregiver	0.06	0.26	0.02	− 0.07	0.02	0.11
No reason or out of the blue	0.03	0.30	− 0.06	0.12	0.19	− 0.04

The factors were interpreted as: (1) Sensory (eigenvalue = 9.60; variance explained = 6.8%; Cronbach’s α = 0.83), which contained items related to sensory hypersensitivity; (2) Cognitive Demand (eigenvalue = 3.79; variance explained = 6.6%; α = 0.79), which consisted of antecedents that might place additional cognitive demand on individuals; (3) Threat to Self (eigenvalue = 1.98; variance explained = 6.6%; α = 0.84), which encompassed antecedents that might be perceived as a threat to the concept of self for individuals; (4) Cross-settings (eigenvalue = 1.86; variance explained = 5.2%; α = 0.78), which included a range of settings and people with whom individuals were more likely to experience outbursts; (5) Safety (eigenvalue = 1.54; variance explained = 4.0%; α = 0.68), which consisted of settings and people associated with safety; (6) States (eigenvalue = 1.35; variance explained = 3.3%; α = 0.68), which included the physiological states, such as tiredness and hunger or thirst.

### Contextual clusters

#### Clusters based on refined factor scores

**Figure 1:**
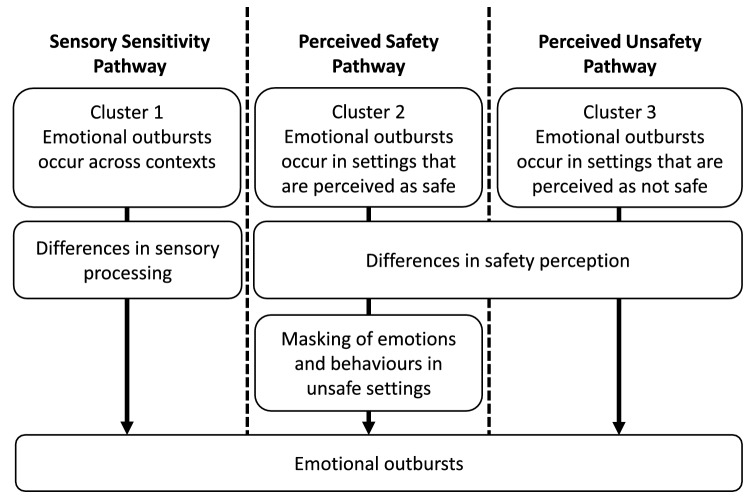
Summary of the description and interpretation of the contextual pathways of emotional outburst, corresponding to the three identified clusters.

Responses were classified into clusters using refined factor scores, which were standardized around means of 0. A three-cluster solution with distinct and interpretable clusters emerged from hierarchical clustering. *K*-means clustering with *k* = 3 provided additional support for this cluster structure, as cluster membership was maintained across clustering methods for 220 participants (82.1%; Cohen’s κ = 0.73, 95% CI [0.66, 0.80]). Based on the results from the cluster analysis, the clusters were ascribed the following labels: (1) Sensory Sensitivity; (2) Perceived Safety; (3) Perceived Unsafety (Fig. 1).

**Figure 2:**
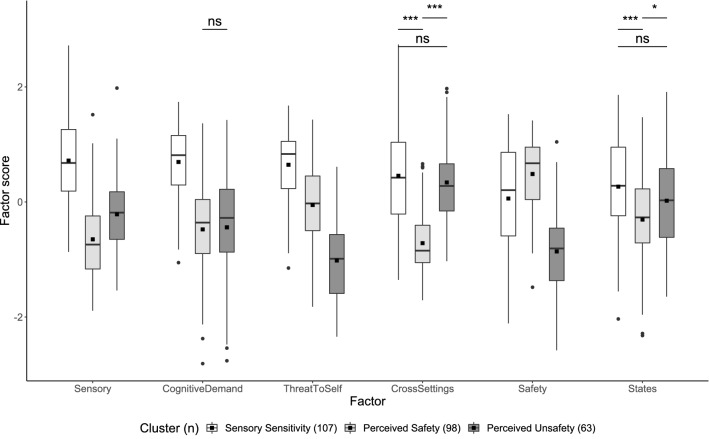
Pairwise comparisons of factor scores for the k-means three-cluster solution. Boxplots show mean (black squares) median (horizontal bar), interquartile range (box), range (whiskers) and outliers (circles). All outliers were included in analyses. Unless otherwise specified, all pairwise comparisons within each factor were significant at *p* < 0.001, adjusted with Tukey’s method. Ns not significant; *p < 0.05.

Comparing the mean factor scores between the three clusters revealed a significant difference (Pillai Trace = 0.651, *F*(6, 261) = 81.034, *p* < 0.001, ω^2^ = 0.642, 95% CI [0.559, 0.700]). Subsequent univariate tests indicated that mean scores for all six factors significantly differed between the three clusters (Table 2). Most post-hoc pairwise comparisons demonstrated significant differences (Fig. 2; Supplementary Table S2). The mean factor scores of the Sensory Sensitivity cluster were generally greater than those of the Perceived Safety and Perceived Unsafety clusters. The Perceived Safety cluster was characterized by a greater mean score in the Safety factor compared to other clusters. The mean scores of the Perceived Unsafety cluster in the Cross-settings and States factors were comparable to the Sensory Sensitivity cluster, and greater than those of the Perceived Safety cluster.

**Table 2:** Univariate comparisons of refined factor scores for the k-means three-cluster solution.

Factor	Cluster Mean (*SD*)			Welch’s *F*	ω^2^	95% CI	Post-hoc summary^a^
	SS (*n* = 107)	PS (*n* = 98)	PU (*n* = 63)				
Sensory	0.72 (0.75)	− 0.65 (0.67)	− 0.21 (0.65)	*F*(2, 161) = 97.0***	0.417	[0.252, 0.561]	1 > 3 > 2
Cognitive demand	0.70 (0.59)	− 0.48 (0.76)	− 0.44 (0.89)	*F*(2,143) = 92.5***	0.406	[0.231, 0.558]	1 > 2, 3
Threat to self	0.65 (0.59)	− 0.05 (0.74)	− 1.02 (0.74)	*F*(2, 149) = 118.6***	0.467	[0.297, 0.608]	1 > 2 > 3
Cross-settings	0.46 (0.90)	− 0.72 (0.53)	0.34 (0.71)	*F*(2, 150) = 91.8***	0.404	[0.234, 0.553]	1, 3 > 2
Safety	0.06 (0.88)	0.49 (0.61)	− 0.86 (0.72)	*F*(2, 155) = 75.1***	0.356	[0.190, 0.508]	2 > 1 > 3
States	0.27 (0.84)	− 0.31 (0.79)	0.02 (0.88)	*F*(2, 154) = 12.7***	0.080	[0.003, 0.209]	1, 3 > 2

SS, Sensory Sensitivity; PS, Perceived Safety; PU, Perceived Unsafety. *p* and confidence intervals adjusted with Bonferroni correction.

^a^Pairwise Games-Howell tests adjusted with Tukey’s method. *** *p* < 0.001.

Differences in demographics between clusters were assessed with ANOVA and χ^2^ tests for variables with sufficient data (Table 3). No difference was found between the clusters in terms of age and gender of the young person, access to medication, access to support, or diagnoses of specific learning difficulties, anxiety, or depression. Exposure to early trauma was more associated with the Sensory Sensitivity and Perceived Safety clusters than the Perceived Unsafety cluster. Diagnoses of ASD, attention deficit hyperactivity disorder, or sensory processing difficulties were more associated with the Sensory Sensitivity cluster. A diagnosis of intellectual disability was more associated with the Perceived Unsafety cluster. It is important to note that despite these diagnostic associations for each cluster, not all individuals with a given diagnosis were exclusively classified to the associated cluster (e.g., there were individuals with sensory processing difficulties in all three clusters), indicating that diagnosis was not the sole determinant of cluster membership. Whilst other diagnoses could not be reliably compared, individuals who shared these diagnoses were also distributed across the three clusters.

**Table 3:** Demographics by cluster for the k-means three-cluster solution using refined factor scores.

Variable	Cluster			Statistic	Effect size^a^	95% CI	Post-hoc summary^b^
	SS	PS	PU				
*N*	107	98	63				
**Age**							
Mean	13.0	13.5	14.4	*F*(2, 150) = 1.25	0.002	[0, 0.023]	
*SD*	4.9	5.1	5.9				
**Gender (%)**				χ^2^ (2) = 4.37^c^	0.128	[0.034, 0.264]	
Male	68.2	54.1	57.1				
Female	31.8	44.9	42.9				
Non-binary	0	1.0	0				
**Diagnosis (%)**^**d**^							
ID	20.6	26.5	46.0	χ^2^ (2) = 12.93**	0.220	[0.112, 0.357]	3 > 1
LD	17.8	10.2	6.3	χ^2^ (2) = 5.42	0.142	[0.052, 0.258]	
ADHD	35.5	15.3	15.9	χ^2^ (2) = 14.29***	0.231	[0.115, 0.347]	1 > 2, 3
ASD	57.9	36.7	54.0	χ^2^ (2) = 9.94**	0.193	[0.090, 0.319]	1 > 2
Anxiety	43.9	36.7	25.4	χ^2^ (2) = 5.85	0.148	[0.047, 0.269]	
Depression	9.3	8.2	6.3	χ^2^ (2) = 0.47	0.042	[0.017, 0.174]	
SPD	16.8	5.1	3.2	χ^2^ (2) = 12.00**	0.212	[0.102, 0.330]	1 > 2, 3^e^
**Medication (%)**							
Yes	28.0	20.4	31.7	χ^2^ (2) = 2.94	0.105	[0.029, 0.238]	
**Access to support (%)**							
Yes	55.1	45.9	41.3	χ^2^ (2) = 3.02	0.107	[0.031, 0.240]	
**Trauma (%)**^**f**^							
Yes	26.2	35.7	15.9	χ^2^ (2) = 13.38**	0.302	[0.172, 0.467]	1, 2 > 3

SS, Sensory Sensitivity; PS, Perceived Safety; PU, Perceived Unsafety; ID, intellectual disability; LD, specific learning difficulties; ASD, autism spectrum disorder; ADHD, attention deficit hyperactive disorder; SPD, sensory processing disorder/difficulties.

^a^ω^2^ for ANOVA and Cramer’s V for χ^2^ tests.

^b^Pairwise χ^2^ tests adjusted with Bonferroni correction.

^c^Non-binary response excluded for χ^2^ test on gender.

^d^Percentage of individuals in each cluster with a given diagnosis. Each caregiver could indicate more than one diagnosis for the multiple-choice question in the survey, so diagnoses were not mutually exclusive and individuals with co-occurring conditions were included in the percentages. Other diagnoses were not included due to insufficient endorsement for statistical comparisons.

^e^Pairwise comparison between the Perceived Safety and Perceived Unsafety clusters not conducted due to insufficient data. ** *p* < 0.01; *** *p* < 0.001.

^f^Percentage of caregivers in each cluster who indicated that their child or young person has experienced early traumatic or adverse events. The proportion of individuals in each cluster for whom trauma data were available for χ^2^ analysis (i.e., selected either *Yes* or *No*) were: 43.9% of the Sensory Sensitivity cluster; 61.2% of the Perceived Safety cluster; 63.5% of the Perceived Unsafety cluster.

Consistent with the higher proportion of autistic individuals in the Sensory Sensitivity and the Perceived Unsafety clusters, scores on the Social Communication Questionnaire^47^ (SCQ) were significantly higher in these clusters compared to the Perceived Safety cluster across all domains and total score (Supplementary Tables S3 and S4).

#### Clusters based on non-refined factor scores

**Figure 3:**
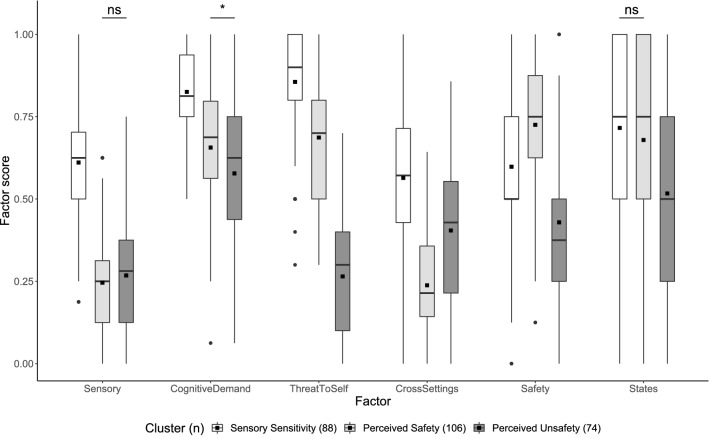
Pairwise comparisons of non-refined factor scores for the k-means three-cluster solution. Boxplots show mean (black squares) median (horizontal bar), interquartile range (box), range (whiskers) and outliers (circles). All outliers were included in analyses. Unless otherwise specified, all pairwise comparisons within each factor were significant at *p* < 0.001, adjusted with Tukey’s method. Ns not significant; *p < 0.05.

To further validate the three-cluster structure obtained from clustering refined factor scores, responses were classified independently based on non-refined factor scores via *k*-means clustering. The two separate cluster solutions using either refined or non-refined factor scores demonstrated agreement for 219 responses (81.7%; Cohen’s κ = 0.72; 95% CI [0.65, 0.79]). There was a significant difference in non-refined factor scores between the three clusters (Pillai Trace = 0.558, *F*(6, 261) = 54.991, *p* < 0.001, ω^2^ = 0.547, 95% CI [0.451, 0.615]). When explored with subsequent univariate and pairwise comparisons, the differences in non-refined factor scores were largely congruent with those found in refined scores (Fig. 3; Table 4 and Supplementary Table S5).

**Table 4:** Univariate comparisons of non-refined factor scores for the *k*-means three-cluster solution.

Factor	Cluster mean (*SD*)			Welch’s *F*	ω^2^	95% CI	Post-hoc summary^a^
	SS (*n* = 88)	PS (*n* = 106)	PU (*n* = 74)				
Sensory	0.61 (0.18)	0.25 (0.17)	0.27 (0.17)	F(2, 166) = 118***	0.466	[0.305, 0.600]	1 > 2, 3
Cognitive demand	0.83 (0.13)	0.66 (0.18)	0.58 (0.21)	F(2, 160) = 49.6***	0.266	[0.115, 0.420]	1 > 2 > 3
Threat to self	0.86 (0.18)	0.69 (0.18)	0.26 (0.19)	F(2, 168) = 217***	0.617	[0.477, 0.723]	1 > 2 > 3
Cross-settings	0.56 (0.21)	0.24 (0.15)	0.40 (0.21)	F(2, 153) = 77.3***	0.363	[0.195, 0.515]	1 > 3 > 2
Safety	0.60 (0.25)	0.73 (0.20)	0.43 (0.22)	F(2, 165) = 42.8***	0.238	[0.095, 0.390]	2 > 1 > 3
States	0.72 (0.27)	0.68 (0.26)	0.52 (0.30)	F(2, 164) = 10.6***	0.067	[0.000, 0.185]	1, 2 > 3

SS, Sensory Sensitivity; PS, Perceived Safety; PU, Perceived Unsafety. *p* and confidence intervals adjusted with Bonferroni correction.

^a^Pairwise Games-Howell tests adjusted with Tukey’s method. ***p < 0.001.

## Discussion

The primary aim of this article was to establish some of the potential contextual pathways of emotional outbursts, primarily in young people with neurodevelopmental disorders and in young people who have experienced early trauma. This was achieved by first extracting and then clustering salient factors related to antecedents and setting events from items within the Emotional Outburst Questionnaire. The resulting three-cluster solution was robust across clustering methods and factor calculation methods. As the non-refined scores are less sample-dependent, this factor calculation method offers generalizability and additional utility, allowing for future classification of new responses^50^. Individuals within the three clusters exhibited unique patterns of contexts in which outbursts occur. The potential contextual pathways and associated mechanisms of emotional outbursts represented by the clusters are presented in Fig. 1.

The Sensory Sensitivity pathway consisted of high scores across contextual factors, indicating that young people within this cluster frequently experienced emotional outbursts across most antecedents and setting events. Accounting for the high Sensory factor scores, it is possible that outbursts for individuals within this cluster may be underpinned by differences in sensory processing, as the additional demands from sensory stimuli may interfere with the cognitive and emotional resources required to mitigate outbursts. This difference in sensory processing may underlie emotional outbursts across other seemingly unrelated contexts for young people in this cluster, as background sensory stimuli may hinder their ability to respond effectively to the contexts in question.

In prior studies, autistic people and their caregivers have described differences in sensory processing as contributing to both anxiety and subsequent meltdowns, demonstrating the capacity for sensory stimuli to act as antecedents of outbursts^5,14,15^. Furthermore, atypical sensory processing in autistic children was associated with 1) increased physiological arousal within a social interaction paradigm, which was suggested to represent an increase in perceived stress, and 2) stress in daily life of the children, as reported by caregivers^16^. These associations indicate that for people with atypical sensory processing, background sensory stimuli may be stressors that could act as setting events and increase the likelihood for antecedents to lead to outbursts.

The current literature has primarily focused on the symptomatology and aetiology of atypical sensory processing within the context of autism (e.g., ref.^17^). Regarding individuals with other neurodevelopmental disorders, atypical sensory processing has been documented in children with ADHD^18^, across different genetic syndromes (e.g., ref.^19^), and in children who have experienced maltreatment^20^. Overall, the potential role of atypical sensory processing in emotional outbursts has received little attention. However, it is conceivable that atypical sensory processing may be involved in outbursts for young people in the Sensory Sensitivity cluster, and that this pathway may be transdiagnostic, as the demographic variables associated with this cluster, namely exposure to trauma, diagnoses of ASD, ADHD, or sensory processing difficulties, have been linked to atypical sensory processing.

Individuals in the Perceived Safety pathway were characterized by high Safety scores and low Cross-settings scores, suggesting that emotional outbursts were more likely to occur in environments perceived to be safe. This pattern of contexts has been observed by Cressey et al.^9^ in interviews with caregivers of individuals with Lowe syndrome, who reported that no outbursts occurred outside the home. In the wider context of challenging behaviours, caregivers, whose children were autistic and had at least one concurrent externalizing disorder, rated their children’s challenging behaviours as more severe compared to teachers^56^. A potential explanation for this discrepancy between informants may be the context dependence of challenging behaviours that is reflected in young people in the Perceived Safety cluster.

A possible mechanism for emotional outbursts in the Perceived Safety cluster may be related to the *generalized unsafety theory of stress*, which posits that when perceived safety is low, individuals exhibit a default stress response driven by the intolerance of uncertainty about safety, even in the absence of explicit stressors^57^. When perceived safety is high, the theory suggests that top-down control is exerted to efficiently inhibit this default stress response^57^. Individuals in the Perceived Safety cluster may perceive environments as less safe and therefore experience more distress in these environments. Indeed, the environmental influence of childhood adversity on safety perception may explain the association of exposure to early trauma with the Perceived Safety cluster^46^. More critically however, individuals in this cluster may be masking their default stress response in such environments. This process of masking is present in the general population (e.g., ref.^58^), but it has largely been explored in the context of camouflaging autistic traits. For instance, autistic individuals reported increased likelihood to camouflage in social environments perceived to be unsafe, such as when people other than close friends and family were present^59,60^. Furthermore, individuals may be motivated to suppress distress that could manifest as emotional outbursts to maintain social desirability amongst peers^61^. For individuals who have experienced childhood adversity or trauma, the motivation to suppress distress and maintain social desirability may be further exacerbated by heightened feelings of shame, which may account for the association of this cluster with exposure to early trauma^62^. When considering the consequences of camouflaging, autistic individuals commonly described the process as exhausting, and successful camouflaging for young people meant that teachers were often unaware of the difficulties that students were facing^59,60^. This “bottling up” of distress when in unsafe environments, which has been anecdotally reported by caregivers of individuals who experience outbursts, may subsequently manifest as emotional outbursts when individuals return to a safe environment, where individuals are not actively suppressing their distress. Additionally, the exhaustion associated with masking may interfere with the ability for individuals to exert top-down control over their default stress response once they return to a safe environment, thus increasing the potential for antecedents to cause outbursts. This pathway may account for the higher proportion of individuals who have experienced traumatic or adverse events within the Perceived Safety cluster, who are more likely to have differences in safety perception^46^.

Young people in the Perceived Unsafety pathway appeared to have a specific difficulty with environments that were not perceived to be safe, suggested by the combination of high Cross-settings and low Safety factor scores. It is important to note that items related to familiar environments loaded strongly onto the Cross-settings factor alongside items related to unsafe or unfamiliar environments, so outbursts for individuals within this cluster were not dependent on environmental novelty. Moreover, although these items may be diametrically opposed, they are not mutually exclusive, as both types of setting events can contribute to the outbursts of a given individual. Due to the relatively high incidence of intellectual disability within the Perceived Unsafety cluster, one may be tempted to adopt a functional perspective when considering the aetiology of outbursts for individuals in this cluster, as challenging behaviours have been argued to serve communicative functions, especially for individuals with intellectual disability, who may have impaired communication ability^27^. However, the extensive body of work examining challenging behaviours in people with intellectual disabilities from a functional perspective has highlighted demand avoidance, access to preferred items or events, and access to social attention as being the primary motivators (establishing operations) for behaviour^28^. Presently, demand avoidance and access to preferred items or events were comprised within the factor labelled Cognitive Demand, for which the mean score for the Perceived Unsafety cluster was relatively low. Furthermore, the item relevant to access to social attention was not included in any of the derived factors. However, from a functional perspective, one might expect these items to feature more prominently within the Perceived Unsafety cluster.

In contrast, the pattern of relevant contexts for individuals in the Perceived Unsafety cluster appears to be consistent with the generalized unsafety theory of stress as described above^57^. It is possible that due to differences in safety perception or inhibition of the default stress response, young people in this cluster may be more intolerant of uncertainty about safety and perceive more environments as unsafe. Unlike individuals in the Perceived Safety cluster, individuals in the Perceived Unsafety cluster may not be actively suppressing their default stress response in these unsafe environments, which may be a consequence of comparatively lower motivation and/or lower ability to suppress such distress. The experience of stress or anxiety across environments with low perceived safety may consequently increase the individuals’ susceptibility to emotional outbursts within these environments. Indeed, several neurodevelopmental disorders associated with outbursts have been linked to heightened intolerance of uncertainty (e.g., refs.^11,21^), which would support the possibility of such differences in safety perception being a transdiagnostic mechanism for emotional outbursts.

Mechanistically, the differences in safety perception may arise from disrupted associative learning. Such a cognitive impairment has been demonstrated in individuals with intellectual disability, in terms of differences in association retention in adults with intellectual disability compared to typically developing controls^63^, and in terms of differences in profiles of associative memory between individuals with Down syndrome and Williams syndrome^64^. From a neurobiological perspective, the potential failure to fully inhibit the default stress response in a safe environment may also account for the association of this cluster with intellectual disability, as differences in prefrontal regions have been observed in individuals with intellectual disability (e.g., ref.^65^).

According to the proposed pathways, the three clusters appear to be characterized by sensitivity to specific setting events, during which individuals may be less able to cope with antecedents. Although distinct diagnostic factors were associated with each cluster, it should be emphasized that the differences proposed to underlie each pathway are not exclusive to the identified diagnoses. The lack of clusters characterized by specific antecedents suggests that antecedents may be secondary to setting events, when considering the framework of contextual pathways. It is possible that setting events and antecedents could be organized into a hierarchy, in which the first level is determined by setting events (Sensory Sensitivity, Perceived Safety, and Perceived Unsafety), followed by subdivisions of the pathways according to antecedents. As with the sensitivity to specific setting events, differences in emotion regulation or expression may lead to individuals struggling with specific antecedents (e.g., changes to routines). However, it is possible that setting events could sufficiently hinder the ability for some individuals to regulate their emotions, such that any additional demand, regardless of susceptibility to specific antecedents, could lead to outbursts.

Attention should be drawn to additional findings that were beyond the original aims of this study. The concern expressed by stakeholders over the use of the term “temper outbursts” during questionnaire development should be emphasized, as this terminology, along with “tantrums” has been frequently used within the literature of challenging behaviours in individuals with neurodevelopmental disorders. However, many caregivers found these terms to be inappropriate because the terms imply that 1) individuals are in control of their outbursts; and 2) outbursts are related mostly to anger. Therefore, it is of critical importance for researchers and professionals to recognize and respect the preferences of families and communities to avoid perpetuating stigma and inaccurate representations of emotional outbursts in people with neurodevelopmental disorders. It should be further noted that the term “meltdowns” was acceptable and sometimes preferred by families. Overall, it would be valuable for future work to evaluate the preferences of families and individuals on outburst terminology more systematically to provide further insight and guidance for researchers and professionals.

Nearly half of the caregivers asked about early traumatic or adverse events reported that their children had been exposed to early trauma. Whilst this may have been influenced by some sampling bias, most recruitment channels used to promote this study were not oriented to trauma-affected individuals. Thus, the relationship between trauma and emotional outbursts should be further explored and accounted for in future work.

Finally, it should be noted that half of the caregivers in this study received no formal support for emotional outbursts. Furthermore, even in families who had access to support, only half reported that the resources were effective. Whilst there is undoubtedly sampling bias that would influence the proportion of responses observed, it is evident that current support systems are limited in both availability and suitability. Therefore, the accessibility and scalability of future interventions and support should be considered to maximize the impact on individuals and families. It is possible that the degree of support accessed may have influenced a caregiver’s ability to identify and accurately report the contexts of emotional outbursts within the present study. Whilst this factor should be considered and potentially controlled for in future work, it would be worthwhile to additionally explore how widespread support could be provided to families to enable more accurate identification and reporting of outburst characteristics.

The primary limitation for this study is the likelihood that not all contextual pathways have been identified, as more uncommon pathways might be overshadowed by one of the three clusters. Furthermore, the low number of responses collected from families with some genetic syndromes or conditions not typically associated with emotional outbursts (e.g., eating disorders) constrained the ability to explore syndrome- or disorder-specific differences in cluster membership. Indeed, our understanding of the relationship between emotional outbursts and many of these diagnoses is limited due to the lack of prior research in these areas. However, the inclusion of individuals with a wide range of diagnoses in the present study may provide the necessary impetus for future work to explore and characterise specific diagnostic differences. A further limitation is that an individual’s outbursts may be related to more than one pathway (e.g., for some autistic individuals, who may be sensitive to both sensory stimuli and the safety of the environment), which was unaccounted for with the current classification method, but the use of more sophisticated clustering algorithms (e.g., subspace multi-clustering methods; for review, see ref.^66^) may overcome this limitation. Lastly, the analysis was based on responses to an author-derived measure, which lacked previous validation or standardisation of the constructs being measured. Despite this limitation, the use of this measure was necessary, as there was a distinct lack of measures from the existing literature that would have been appropriate for the present aims. Moreover, the Emotional Outburst Questionnaire was based on previously validated measures and some aspects of its validity were demonstrated in the present study.

In terms of future directions, this study provides the foundation for a transdiagnostic approach to characterize and explore the contextual pathways of emotional outbursts. It is possible that the distribution of individuals within each contextual cluster may vary across genetic syndromes and other diagnoses, which may provide further insight into the mechanisms underlying each pathway. Furthermore, the proposed pathways warrant further investigation, in terms of assessing their face validity to families, and verifying and expanding on the mechanisms associated with each pathway. Ultimately, operating under this framework may facilitate the development of pathway-specific intervention strategies. For example, interventions targeting outbursts in the Perceived Safety pathway may include components that enable individuals to self-regulate in unsafe environments to prevent the build-up of distress and additionally consider the underlying motivations for masking.

The current article presented the Emotional Outburst Questionnaire as a new tool to characterize and classify emotional outbursts in terms of related contexts in children and young people with neurodevelopmental disorders or early traumatic experiences. Three potential contextual pathways and their associated mechanisms were established. The three pathways were proposed to be related to specific environmental sensitivities of individuals and their response to these aspects of the environment, which might limit the ability for individuals to regulate their emotions and behaviours in response to antecedents.

## Supplementary Information

Supplementary Information.

## Data Availability

The data analysed in this study can be found at https://osf.io/2j47e/.

## Code availability

The computer code used to generate the results can be found at https://osf.io/2j47e/.
