# Supplementary material for: Establishing the transdiagnostic contextual pathways of emotional outbursts
Source: Sci Rep. 2022 May 6;12:7414. doi: 10.1038/s41598-022-11474-4 (PMC9076826; doi:10.1038/s41598-022-11474-4)
Supplement: Supplementary file 1 — Supplementary Information. [file 41598_2022_11474_MOESM1_ESM.pdf]

Supplementary Information

Supplementary Methods

The Emotional Outburst Questionnaire was developed to transdiagnostically measure the characteristics of outbursts in young people (aged 6-25 years) via informant report. Characteristics of emotional outbursts have been commonly assessed using various informant-report semi-structured interview schedules and questionnaires in typically developing individuals (e.g., refs. [1,2]) and individuals with specific neurodevelopmental disorders[3–7]. These existing measures of outbursts informed the development of the Emotional Outburst Questionnaire (Supplementary Table S6). Some measures were directly related to emotional outbursts, whilst others considered the wider context of challenging behaviours, of which outbursts are an example.

Broad categories of outburst characteristics were identified, resembling the categories from studies of outbursts in people with Prader-Willi syndrome[5,6]. This article specifically focused on the contextual categories of Setting events and Antecedents (Supplementary Table S7), as the development and analysis of the other sections of the Emotional Outburst Questionnaire will be reported in a subsequent article. Items pertaining to emotional outbursts from the existing measures were compiled into an exhaustive list. Related items were combined to reduce the number of items.

The first author collected feedback from caregivers and professionals on a prototype version of the Emotional Outburst Questionnaire. Stakeholders were recruited based on recommendations from support groups, and through connections with the research team. Twenty caregivers of young people (mean age 11.5 years; standard deviation (*SD*) = 4.1; range = 4–21; 17 male and 7 female; see Supplementary Table S8 for diagnostic information) exhibiting difficulties with outbursts provided feedback in focus group or one-on-one interview settings. Further feedback was obtained via interviews or written communications from four professionals with experience in working with young people with neurodevelopmental disorders across various settings (one special schoolteacher,

three clinical psychologists). Interviews and focus groups were recorded and transcribed, supplemented by notes made during the sessions.

Stakeholders assessed the prototype questionnaire in terms of the relevance of each item, the comprehensibility of the questionnaire, and the comprehensiveness of the measure. Specific focus was directed to the wording of instructions and items, and the lists of examples at the end of items to ensure that key examples were included. The interpretations of rating scales were assessed to ensure consistency in interpretation across individuals. Following a round of revision, stakeholders were invited to provide further feedback on the revised version. One stakeholder provided additional comments, which were incorporated into the final design.

The final version of the questionnaire involved the following amendments: addition of a suitable recall period, addition and removal of items, rearrangement of item order, and rewording of items and instructions. One of the most significant changes was the transition away from the term "temper outburst" due to its negative connotations and associations with "temper tantrum". Examples of specific changes pertaining to the context of outbursts included additions of the following items: "Specific phobia or fear", "Food-related triggers", and "Not understanding what is going on".

Supplementary Table S1 Diagnostic information of young people whose caregivers completed the survey.

Diagnosis	*n*	%
**Neurodevelopmental**		
Intellectual disability	77	28.7
Specific learning difficulties [a]	33	12.3
Attention deficit hyperactive disorder	63	23.5
Autism spectrum disorder	132	49.3
Cornelia deLange syndrome	15	5.6
CHARGE syndrome	7	2.6
DiGeorge syndrome	2	0.7
Down syndrome	4	1.5
Fragile X syndrome	2	0.7
RASopathies [b]	9	3.4
Prader-Willi syndrome	9	3.4
Tuberous sclerosis complex	6	2.2
Williams syndrome	14	5.2
Klinefelter syndrome	2	0.7
Other genetic disorders	8	3.0
FASD and other prenatal drug exposure	14	5.2
Idiopathic neurodevelopmental disorders	3	1.1
**Psychiatric**		
Anxiety	99	36.9
Attachment difficulties/disorders	19	7.1
Borderline personality disorder	2	0.7
Communication disorders	4	1.5
Demand avoidance	7	2.6
Depression	22	8.2
Disruptive, impulse control, and conduct disorders	7	2.6
Eating disorders	2	0.7
Mood disorders	2	0.7
Obsessive-compulsive disorder	5	1.9
Post-traumatic stress disorder	3	1.1
**Physical**		
Sensory processing disorder/difficulties	25	9.3
Hearing impairments	13	4.9
Visual impairments	3	1.1
Dyspraxia	4	1.5
Epilepsy	5	1.9
Tic disorders	4	1.5
Joint conditions	8	3.0
Other physical conditions	2	0.7

Diagnosis	*n*	%
**Other**		
Other cognitive impairments	1	0.4
Metabolic conditions	1	0.4
Awaiting or undergoing assessment	35	13.1
No diagnosis	12	4.5

FASD, foetal alcohol spectrum disorder.

[a] E.g., dyslexia.

[b] RASopathies include Noonan syndrome, neurofibromatosis type 1, and related disorders.

Supplementary Table S2 Mean differences in pairwise comparisons of refined factor scores for the *k*-means three-cluster solution.

Factor	Mean difference (95% CI)		
	Cluster SS-PS	Cluster SS-PU	Cluster PS-PU
Sensory	-1.37 [-1.60, -1.13] ***	-0.93 [-1.19, -0.67] ***	0.43 [0.18, 0.69] ***
Cognitive Demand	-1.17 [-1.40, -0.95] ***	-1.14 [-1.44, -0.84] ***	0.04 [-0.29, 0.36]
Threat to Self	-0.70 [-0.92, -0.48] ***	-1.66 [-1.92, -1.40] ***	-0.96 [-1.25, -0.68] ***
Cross-settings	-1.17 [-1.41, -0.93] ***	-0.12 [-0.41, 0.18]	1.06 [0.81, 1.30] ***
Safety	0.43 [0.18, 0.67] ***	-0.92 [-1.21, -0.63] ***	-1.35 [-1.61, -1.09] ***
States	-0.57 [-0.84, -0.30] ***	-0.25 [-0.57, 0.08]	0.33[0, 0.65] *

SS, Sensory Sensitivity; PS, Perceived Safety; PU, Perceived Unsafety.

p and confidence intervals adjusted with Tukey's method.

* $p < 0.05$; *** $p < 0.001$.

Supplementary Table S3 Univariate comparisons of Social Communication Questionnaire scores for the k-means three-cluster solution using refined factor scores.

SCQ domain	Cluster Mean (*SD*)			Welch's *F*	ω^2	95% CI	Post-hoc Summary [a]
	SS (*n* = 107)	PS (*n* = 98)	PU (*n* = 63)				
Social	8.10 (3.74)	5.03 (3.91)	8.27 (4.08)	$F(2, 154) = 19.9^{***}$	0.124	[0.038, 0.226]	1, 3 > 2
Communication	6.72 (2.79)	5.04 (3.08)	6.73 (2.28)	$F(2, 166) = 10.3^{***}$	0.065	[0.008, 0.146]	1, 3 > 2
Repetitive	5.36 (2.03)	3.53 (2.40)	5.59 (2.15)	$F(2, 155) = 21.8^{***}$	0.135	[0.045, 0.238]	1, 3 > 2
Total	22.21 (7.50)	14.77 (8.45)	22.32 (6.78)	$F(2, 162) = 26.8^{***}$	0.162	[0.066, 0.267]	1, 3 > 2

SS, Sensory Sensitivity; PS, Perceived Safety; PU, Perceived Unsafety.

*** $p < 0.001$.

[a] Pairwise Games-Howell tests adjusted with Tukey's method.

Supplementary Table S4 Mean differences in pairwise comparisons of Social Communication Questionnaire scores for the k-means three-cluster solution using refined factor scores.

SCQ domain	Mean difference (95% *CI*)		
	Cluster SS-PS	Cluster SS-PU	Cluster PS-PU
Social	-3.07 [-4.34, -1.80] ***	0.17 [-1.32, 1.66]	3.24 [1.70, 4.78] ***
Communication	-1.68 [-2.65, -0.703] ***	0.01 [-0.92, 0.94]	1.69 [0.68, 2.69] ***
Repetitive	-1.84 [-2.58, -1.10] ***	0.22 [-0.57, 1.01]	2.06 [1.20, 2.92] ***
Total	-7.44 [-10.1, -4.79] ***	0.10 [-2.55, 2.76]	7.54 [4.68, 10.4] ***

SS, Sensory Sensitivity; PS, Perceived Safety; PU, Perceived Unsafety.

p and confidence intervals adjusted with Tukey's method.

***** p *< 0.001.*

Supplementary Table S5 Mean differences in pairwise comparisons of non-refined factor scores for the *k*-means three-cluster solution.

Factor	Mean difference (95% *CI*)		
	Cluster SS-PS	Cluster SS-PU	Cluster PS-PU
Sensory	-0.36 [-0.42, -0.31] ***	-0.34 [-0.41, -0.28] ***	0.02 [-0.04, 0.08]
Cognitive Demand	-0.17 [-0.22, -0.12] ***	-0.25 [-0.32, -0.18] ***	-0.08 [-0.15, -0.01] *
Threat to Self	-0.17 [-0.23, -0.11] ***	-0.59 [-0.66, -0.52] ***	-0.42 [-0.49, -0.36] ***
Cross-settings	-0.33 [-0.39, -0.26] ***	-0.16 [-0.24, -0.08] ***	0.17 [0.10, 0.23] ***
Safety	0.13 [0.05, 0.21] ***	-0.17 [-0.26, -0.08] ***	-0.30 [-0.37, -0.22] ***
States	-0.04 [-0.13, 0.05]	-0.20 [-0.31, -0.09] ***	-0.16 [-0.26, -0.06] ***

SS, Sensory Sensitivity; PS, Perceived Safety; PU, Perceived Unsafety.

p and confidence intervals adjusted with Tukey's method.

** p < 0.05; *** p < 0.001.*

Supplementary Table S6 Existing measures that informed the development of the Emotional Outburst Questionnaire.

Measure name	Measure type	Reliability and validity
-[6]	Semi-structured informant interview	Convergent validity: 0.66-1

-[5]	Informant-report questionnaire	Test-retest reliability: 0.52-0.76
Multidimensional Assessment of Preschool Disruptive Behavior – Temper Loss scale[2,8]	Informant-report questionnaire	Internal consistency: 0.97<br>Test-retest reliability: 0.80
Irritability Inventory[9]	Informant-report questionnaire	-
Temper Tantrum Grid[10]	Observational tool	-
-[11]	Observations	-
Autism spectrum disorder – behavior problems for children[12]	Informant-report questionnaire	Inter-informant reliability: 0.49<br>Test-retest reliability: 0.63<br>Internal consistency: 0.90
Contextual Assessment Inventory[13]	Informant-report questionnaire	Inter-informant reliability: 0.28<br>Test-retest reliability: 0.61-0.74<br>Internal consistency: 0.95
Modified Overt Aggression Scale[14]	Informant-report questionnaire	Inter-informant reliability: 0.85-0.94<br>Test-retest reliability: 0.72

Supplementary Table S7 Features of contextual items in the Emotional Outburst Questionnaire.

Characteristic	Description	Number of items	Example item
Setting events	Environmental factors that increase the likelihood of emotional outbursts.	19	A place that makes them feel safe.
Antecedents	Events that directly trigger emotional outbursts.	36	Change in own routine.

Supplementary Table S8 Diagnostic information of individuals whose caregivers contributed to the development of the Emotional Outburst Questionnaire.

Diagnosis	*n*
Intellectual disability	10
ASD	16
ADHD	4
FASD	2
Genetic disorders	3
Under diagnostic assessment	4

ASD, autism spectrum disorder; ADHD, attention deficit hyperactive disorder; FASD, foetal alcohol spectrum disorder.

The Emotional Outburst Questionnaire

The term "*emotional outburst*" refers to a highly emotional or explosive episode, where at least one of the behaviours listed below (items 2-23) is displayed. Emotional outbursts may also be known as "*meltdowns*", "*crisis*", "*behavioural breakdown", "blips*", "*rages*", "*temper outbursts*", "*tantrums*", or "*tempers*".

In this questionnaire, we want you to think about the **most severe** and **least severe** emotional outbursts **within the past month** that the individual you care for has displayed and the characteristics associated with each type of emotional outburst, such as behaviours, frequency, and duration. In terms of the **severity** of emotional outbursts, we are referring to how disruptive and negatively impactful they are to the person and/or those around them **at the time** of the emotional outburst.

If you feel that the **severity** of emotional outbursts is always the same, please answer the questions relating to the **most severe** type. We recognise that some questions may be difficult to answer, as emotional outbursts can vary greatly depending on the context. However, please try to give an average for these questions, as this will help us to better understand emotional outbursts over a range of contexts.

First, we would like you to consider the **most severe** emotional outbursts that the individual you care for has displayed **within the past month**.

1	Please list **up to 20 words** to describe what distinguishes the **most severe** emotional outbursts. *(e.g. physically aggressive, screaming, at least an hour)*	

Please indicate your answer for each of the following items by ticking the appropriate box (☐).

During the **most severe** emotional outbursts, how often does the individual you care for display the following behaviours?		Not applicable/never/rarely (0-3 times out of 10 outbursts)	Sometimes (4-6 times out of 10 outbursts)	Often/always (7-10 times out of 10 outbursts)
2	Behavioural indicators of emotion *(e.g. angry or annoyed facial expressions, crying, signs of distress, whining)*	☐	☐	☐
3	Mild verbal aggression *(e.g. insults, name-calling, screaming, shouting, swearing)*	☐	☐	☐
4	Extreme verbal aggression *(e.g. threats of violence)*	☐	☐	☐

5	Non-speech vocalisations<br>(*e.g. making sounds or noises*)	☐	☐	☐
6	Mild aggression towards property<br>*(e.g. defacing walls, ripping clothing, slamming door, throwing objects down)*	☐	☐	☐
7	Extreme aggression towards property<br>*(e.g. breaking objects, smashing windows, throwing objects dangerously)*	☐	☐	☐
		Not applicable/never/rarely (0-3 times out of 10 outbursts)	Sometimes (4-6 times out of 10 outbursts)	Often/always (7-10 times out of 10 outbursts)
8	Mild physical aggression towards others **without physical injury**<br>*(e.g. biting, grabbing, hitting, kicking, pulling hair, pushing, scratching, spitting, throwing objects at people)*	☐	☐	☐
9	Extreme physical aggression towards others **with physical injury**<br>*(e.g. biting, grabbing, hitting, kicking, pulling hair, pushing, scratching, throwing objects at people)*	☐	☐	☐
10	Mild self-injurious behaviours **without serious injury** (no cuts, bruises, burns, etc)<br>*(e.g. banging head, biting, hitting self, hitting wall, holding breath, picking skin, pulling hair)*	☐	☐	☐
11	Extreme self-injurious behaviours **with serious injury**<br>*(e.g. banging head, biting, hitting self, hitting wall, picking skin, picking rectum, pulling hair)*	☐	☐	☐
		Not applicable/never/rarely (0-3 times out of 10 outbursts)	Sometimes (4-6 times out of 10 outbursts)	Often/always (7-10 times out of 10 outbursts)

12	Talking to self & others<br>*(e.g. agitated talking, repetitive speech, self-deprecating speech)*	☐	☐	☐
13	Increased motor activity<br>*(e.g. flailing arms, non-directed kicking, pacing, repetitive behaviours, rushing about, stamping feet, tics)*	☐	☐	☐
14	Increased physiological arousal<br>*(e.g. red face, salivating, sweating)*	☐	☐	☐
15	Avoidance<br>*(e.g. dropping to floor, going to room, leaving situation, running away)*	☐	☐	☐
16	Removing items of clothing	☐	☐	☐
17	Defecation or urination	☐	☐	☐
18	Contextually inappropriate sexual behaviours	☐	☐	☐
19	Ignoring or not talking to certain people	☐	☐	☐
20	Not reacting to things going on around them	☐	☐	☐
		Not applicable/never/rarely (0-3 times out of 10 outbursts)	Sometimes (4-6 times out of 10 outbursts)	Often/always (7-10 times out of 10 outbursts)
21	Food-related behaviours<br>(*e.g. grabbing, pleading for, seeking, or stealing food*)	☐	☐	☐
22	Making themselves sick<br>(*e.g. retching or vomiting*)	☐	☐	☐
23	Unusual behaviours	☐	☐	☐

Advice and support around protecting the individual you care for is available via the NSPCC, Papyrus, or Samaritans.

Website: nspcc.org.uk
Email: help@nspcc.org.uk
Telephone: 0808 800 5000

Website: papyrus-uk.org
Email: pat@papyrus-uk.org
Telephone: 0800 068 4141

Website: samaritans.org
Email: jo@samaritans.org
Telephone: 116 123

24	How often do the **most severe** emotional outbursts occur?	☐ Never	☐ Less than once a month	☐ Once a month	☐ 2-3 times a month	☐ Once a week	☐ 2-3 times a week	☐ Once a day	☐ More than once a day

25	How long do the **most severe** emotional outbursts last?	☐ Less than 5 minutes	☐ 5-15 minutes	☐ 15-30 minutes	☐ 30 minutes to 1 hour	☐ 1-2 hours	☐ 2 hours to a day	☐ A day or more
26	How angry or upset does the person get during the **most severe** emotional outbursts?	☐ 1 Not angry or upset at all	☐ 2	☐ 3	☐ 4	☐ 5	☐ 6	☐ 7 As angry or upset as I have ever seen them

27	Compared to baseline behaviour, how much eye contact does the person seek from you during the **most severe** emotional outbursts?	☐ Less than baseline	☐ Same as baseline	☐ More than baseline

28	How long does it take for the person to recover from the **most severe** emotional outbursts (i.e. from the end of emotional outburst behaviours to when behaviour is back to normal)?	☐ Less than 5 minutes	☐ 5-15 minutes	☐ 15-30 minutes	☐ 30 minutes to 1 hour	☐ 1-2 hours	☐ 2 hours to a day	☐ A day or more

Now, we would like you to consider the **least severe** emotional outbursts that the individual you care for has displayed **within the past month**, that nevertheless disrupt and negatively impact them and/or those around them. We are referring to episodes that are different from the person's normal or baseline behaviour. The term "*emotional outburst*" refers to a highly emotional or explosive

episode, where at least one of the behaviours listed above (items 2-23) is displayed. Emotional outbursts may also be known as “*meltdowns*”, “*crisis*", “*behavioural breakdown”, “blips*”, “*rages*”, “*temper outbursts*”, “*tantrums*”, or “*tempers*”.

If you feel that the **severity** of emotional outbursts is always the same, please tick the ‘Not applicable’ box below and continue from item 57.

29	Please list **up to 20 words** to describe what distinguishes the **least severe** emotional outbursts. *(e.g. crying, red face, no more than 5 minutes)*	☐ Not applicable

Please indicate your answer for each of the following items by ticking the appropriate box (☐).

During the **least severe** emotional outbursts, how often do they display the following behaviours?	Not applicable/never/rarely (0-3 times out of 10 outbursts)	Sometimes (4-6 times out of 10 outbursts)	Often/always (7-10 times out of 10 outbursts)
30 Behavioural indicators of emotion *(e.g. angry or annoyed facial expressions, crying, signs of distress, whining)*	☐	☐	☐
31 Mild verbal aggression *(e.g. insults, name-calling, screaming, shouting, swearing)*	☐	☐	☐
32 Extreme verbal aggression *(e.g. threats of violence)*	☐	☐	☐
33 Non-speech vocalisations (*e.g. making sounds or noises*)	☐	☐	☐
34 Mild aggression towards property *(e.g. defacing walls, ripping clothing, slamming door, throwing objects down)*	☐	☐	☐
35 Extreme aggression towards property *(e.g. breaking objects, smashing windows, throwing objects dangerously)*	☐	☐	☐
36 Mild physical aggression towards others **without physical injury** *(e.g. biting, grabbing, hitting, kicking,*	☐	☐	☐

	pulling hair, pushing, scratching, spitting, throwing objects at people)			
		Not applicable/never/rarely (0-3 times out of 10 outbursts)	Sometimes (4-6 times out of 10 outbursts)	Often/always (7-10 times out of 10 outbursts)
37	Extreme physical aggression towards others **with physical injury** *(e.g. biting, grabbing, hitting, kicking, pulling hair, pushing, scratching, throwing objects at people)*	☐	☐	☐
38	Mild self-injurious behaviours **without serious injury** (no cuts, bruises, burns, etc) *(e.g. banging head, biting, hitting self, hitting wall, holding breath, picking skin, pulling hair)*	☐	☐	☐
39	Extreme self-injurious behaviours **with serious injury** *(e.g. banging head, biting, hitting self, hitting wall, picking skin, picking rectum, pulling hair)*	☐	☐	☐
40	Talking to self & others *(e.g. agitated talking, repetitive speech, self-deprecating speech)*	☐	☐	☐
41	Increased motor activity *(e.g. flailing arms, non-directed kicking, pacing, repetitive behaviours, rushing about, stamping feet, tics)*	☐	☐	☐
42	Increased physiological arousal *(e.g. red face, salivating, sweating)*	☐	☐	☐
43	Avoidance *(e.g. dropping to floor, going to room, leaving situation, running away)*	☐	☐	☐
		Not applicable/never/rarely (0-3 times out of 10 outbursts)	Sometimes (4-6 times out of 10 outbursts)	Often/always (7-10 times out of 10 outbursts)
44	Removing items of clothing	☐	☐	☐

45	Defecation or urination	☐	☐	☐
46	Contextually inappropriate sexual behaviours	☐	☐	☐
47	Ignoring or not talking to certain people	☐	☐	☐
48	Not reacting to things going on around them	☐	☐	☐
49	Food-related behaviours (*e.g. grabbing, pleading for, seeking, or stealing food*)	☐	☐	☐
		Not applicable/never/rarely (0-3 times out of 10 outbursts)	Sometimes (4-6 times out of 10 outbursts)	Often/always (7-10 times out of 10 outbursts)
50	Making themselves sick (*e.g. retching or vomiting*)	☐	☐	☐
51	Unusual behaviours	☐	☐	☐

Advice and support around protecting the individual you care for is available via the NSPCC, Papyrus, or Samaritans.

Website: nspcc.org.uk
Email: help@nspcc.org.uk
Telephone: 0808 800 5000

Website: papyrus-uk.org
Email: pat@papyrus-uk.org
Telephone: 0800 068 4141

Website: samaritans.org
Email: jo@samaritans.org
Telephone: 116 123

52	How often do the **least severe** emotional outbursts occur?	☐ Never	☐ Less than once a month	☐ Once a month	☐ 2-3 times a month	☐ Once a week	☐ 2-3 times a week	☐ Once a day	☐ More than once a day

53	How long do the **least severe** emotional outbursts last?	☐ Less than 5	☐ 5-15 minutes	☐ 15-30 minutes	☐ 30 minut	☐ 1-2 hours	☐ 2 hours	☐ A day or more

		minut es			es to 1 hour		to a day	
54	How angry or upset does person get during the **least severe** emotional outbursts?	☐ 1 Not angry or upset at all	☐ 2	☐ 3	☐ 4	☐ 5	☐ 6	☐ 7 As angry or upset as I have ever seen them
55	Compared to baseline behaviour, how much eye contact does the person seek from you during the **least severe** emotional outbursts?	☐ Less than baseline			☐ Same as baseline		☐ More than baseline	
56	How long does it take for person to recover from the **least severe** emotional outbursts (i.e. from the end of emotional outburst behaviours to when behaviour is back to normal)?	☐ Less than 5 minut es	☐ 5-15 minut es	☐ 15-30 minut es	☐ 30 minut es to 1 hour	☐ 1-2 hours	☐ 2 hours to a day	☐ A day or more

We would like you to consider **in general, all emotional outbursts** the individual you care for has displayed **within the past month**.

Please indicate your answer for each item by ticking the appropriate box (☐).

57	How often do emotional outbursts occur?	☐ Never	☐ Less than once a month	☐ Once a month	☐ 2-3 times a month	☐ Once a week	☐ 2-3 times a week	☐ Once a day	☐ More than once a day

When the individual you care for is in the following places, how often do emotional outbursts occur?		Not applicable/never/rarely (0-3 times out of 10)	Sometimes (4-6 times out of 10)	Often/always (7-10 times out of 10)
58	A place that makes them feel safe	☐	☐	☐
59	A place that makes them feel unsafe	☐	☐	☐
60	A place that they are familiar with *(e.g. at a relative/friend's house)*	☐	☐	☐
61	A place that they are unfamiliar with *(e.g. whilst on holiday away from home)*	☐	☐	☐
62	A place that they feel is private *(e.g. in their room)*	☐	☐	☐
63	A place that they feel is public *(e.g. at a shop)*	☐	☐	☐

When the individual you care for is with the following people, how often do emotional outbursts occur?		Not applicable/never/rarely (0-3 times out of 10)	Sometimes (4-6 times out of 10)	Often/always (7-10 times out of 10)
64	Someone that makes them feel safe (*e.g. a parent/caregiver)*	☐	☐	☐
65	Someone that makes them feel unsafe *(e.g. a dentist)*	☐	☐	☐
66	Someone familiar *(e.g. a teacher)*	☐	☐	☐
67	Someone unfamiliar *(e.g. a cashier at a shop)*	☐	☐	☐

68	Someone they like	☐	☐	☐
69	Someone they dislike	☐	☐	☐
70	Someone they are jealous of	☐	☐	☐

When the individual you care for is in the following states, how often do emotional outbursts occur?		Not applicable/never/rarely (0-3 times out of 10)	Sometimes (4-6 times out of 10)	Often/always (7-10 times out of 10)
71	Tired	☐	☐	☐
72	Hungry or thirsty	☐	☐	☐
73	Consumed too much of one type of food or drink *(e.g. caffeine)*	☐	☐	☐
74	Illness	☐	☐	☐
75	In pain	☐	☐	☐
76	In a bad mood or having a bad day	☐	☐	☐

When the following trigger events occur, how often do they lead to an emotional outburst?		Not applicable/never/rarely (0-3 times out of 10)	Sometimes (4-6 times out of 10)	Often/always (7-10 times out of 10)
77	Planned transition from one activity to another	☐	☐	☐
78	Change in own routine	☐	☐	☐
79	Change in another's routine	☐	☐	☐
80	Change in expectation	☐	☐	☐
81	Being fixated on a thought or idea	☐	☐	☐
82	Specific phobia or fear	☐	☐	☐
83	Food-related triggers	☐	☐	☐
84	Concerns for own property (e.g. losing something or worried about losing something)	☐	☐	☐

		Not applicable/never/rarely (0-3 times out of 10)	Sometimes (4-6 times out of 10)	Often/always (7-10 times out of 10)
85	Not being given or not being able to do something the person wants	☐	☐	☐
86	Having to wait before being given or being able to do something	☐	☐	☐
87	Being asked to do something the person may or may not want to do	☐	☐	☐
88	Doing a boring task	☐	☐	☐
89	Doing a difficult task	☐	☐	☐
90	Doing a repetitive task	☐	☐	☐
		Not applicable/never/rarely (0-3 times out of 10)	Sometimes (4-6 times out of 10)	Often/always (7-10 times out of 10)
91	Doing a new task	☐	☐	☐
92	Under time pressure (e.g. getting ready in the morning)	☐	☐	☐
93	Disagreement with others	☐	☐	☐
94	Being told off, criticised, or accused of making a mistake	☐	☐	☐
95	Being teased	☐	☐	☐
96	Being apart from parent(s)/caregiver	☐	☐	☐
97	Not receiving enough attention or being ignored	☐	☐	☐
98	Receiving too much attention	☐	☐	☐
99	Feeling of being treated unfairly	☐	☐	☐
100	Someone not understanding the individual you care for	☐	☐	☐
101	The individual you care for not understanding someone else	☐	☐	☐
		Not applicable/never/ra	Sometimes (4-6 times out of 10)	Often/always (7-10 times out of 10)

		rely (0-3 times out of 10)		
102	Not understanding what is going on	☐	☐	☐
103	Receiving conflicting information	☐	☐	☐
104	Light is too bright	☐	☐	☐
105	Sudden or loud noises	☐	☐	☐
106	Temperature is too hot or too cold	☐	☐	☐
107	Particular smells or strong smells	☐	☐	☐
108	Touch-related over-sensitivity (e.g. uncomfortable seat or sudden touch)	☐	☐	☐
109	Other sensory-related triggers<br>Specify:	☐	☐	☐
110	Medication side-effect	☐	☐	☐
111	Mood of parent/caregiver	☐	☐	☐
112	No reason/out of the blue	☐	☐	☐
113	How confident are you in your answers above (items 77-112) relating to the triggers that lead to emotional outbursts?	Not confident<br>☐	Quite confident<br>☐	Very confident<br>☐

How successful are the following management strategies in calming emotional outbursts of the individual you care for?		Not applicable/never/rarely (0-3 times out of 10)	Sometimes (4-6 times out of 10)	Often/always (7-10 times out of 10)
114	Physical or verbal comfort	☐	☐	☐
115	Discussion or persuasion	☐	☐	☐

116	Calming or relaxation strategies	☐	☐	☐
117	Giving them what they want	☐	☐	☐
118	Visual aids	☐	☐	☐
119	Punishment or threat of punishment	☐	☐	☐
120	Negotiation	☐	☐	☐
121	Actively ignoring behaviour	☐	☐	☐
122	Moving them or others from situation	☐	☐	☐
123	Distraction	☐	☐	☐
124	Showing empathy	☐	☐	☐

How often does the individual you care for display the following behaviours **after** emotional outbursts?		Not applicable/never/rarely (0-3 times out of 10 outbursts)	Sometimes (4-6 times out of 10 outbursts)	Often/always (7-10 times out of 10 outbursts)
125	Apologising	☐	☐	☐
126	Blaming others	☐	☐	☐
127	Seeking reassurance or comfort	☐	☐	☐
128	Appearing withdrawn	☐	☐	☐
129	Staying in a bad mood	☐	☐	☐
130	Feeling anxious	☐	☐	☐
131	Feeling sad	☐	☐	☐
132	Behaving as if nothing had happened	☐	☐	☐

		Never/rarely (0-3 times out of 10 outbursts)	Sometimes (4-6 times out of 10 outbursts)	Often/always (7-10 times out of 10 outbursts)
133	How often are you there to witness the emotional outbursts when they occur?	☐	☐	☐

References

1. Belden, A. C., Thomson, N. R. & Luby, J. L. Temper Tantrums in Healthy Versus Depressed and Disruptive Preschoolers: Defining Tantrum Behaviors Associated with Clinical Problems. *J. Pediatr.* **152**, 117–122 (2008).
2. Wakschlag, L. S. *et al.* Defining the developmental parameters of temper loss in early childhood: Implications for developmental psychopathology. *J. Child Psychol. Psychiatry Allied Discip.* **53**, 1099–1108 (2012).
3. Beauchamp-Châtel, A., Courchesne, V., Forgeot d'Arc, B. & Mottron, L. Are tantrums in autism distinct from those of other childhood conditions? A comparative prevalence and naturalistic study. *Res. Autism Spectr. Disord.* **62**, 66–74 (2019).
4. Cressey, H., Oliver, C., Crawford, H. & Waite, J. Temper outbursts in Lowe syndrome: Characteristics, sequence, environmental context and comparison to Prader–Willi syndrome. *J. Appl. Res. Intellect. Disabil.* **32**, 1216–1227 (2019).
5. Rice, L. J., Woodcock, K. A. & Einfeld, S. L. The characteristics of temper outbursts in Prader–Willi syndrome. *Am. J. Med. Genet. Part A* **176**, 2292–2300 (2018).
6. Tunnicliffe, P., Woodcock, K., Bull, L., Oliver, C. & Penhallow, J. Temper outbursts in Prader-Willi syndrome: Causes, behavioural and emotional sequence and responses by carers. *J. Intellect. Disabil. Res.* **58**, 134–150 (2014).
7. Tureck, K., Matson, J. L., May, A. & Turygin, N. Externalizing and tantrum behaviours in children with ASD and ADHD compared to children with ADHD. *Dev. Neurorehabil.* **16**, 52–57 (2013).
8. Wakschlag, L. S. *et al.* Advancing a multidimensional, developmental spectrum approach to preschool disruptive behavior. *J. Am. Acad. Child Adolesc. Psychiatry* **53**, 82-96.e3 (2014).
9. Carlson, G. A., Danzig, A. P., Dougherty, L. R., Bufferd, S. J. & Klein, D. N. Loss of Temper and Irritability: The Relationship to Tantrums in a Community and Clinical Sample. *J. Child Adolesc. Psychopharmacol.* **26**, 114–122 (2016).

10. Potegal, M., Carlson, G., Margulies, D., Gutkovitch, Z. & Wall, M. Rages or temper tantrums? The behavioral organization, temporal characteristics, and clinical significance of angry-agitated outbursts in child psychiatry inpatients. *Child Psychiatry Hum. Dev.* **40**, 621–636 (2009).

11. Eisbach, S. S. *et al.* Characteristics of in Preschoolers with Disruptive Behavior. *J. Psychosoc. Nurs. Ment. Health Serv.* **52**, 32–40 (2014).

12. Matson, J. L. & Gonzalez, M. L. *Autism spectrum disorder – behavior problems for children*. (Disability Consultants, LLC, 2007).

13. McAtee, M., Carr, E. G. & Schulte, C. A Contextual Assessment Inventory for Problem Behavior. *J. Posit. Behav. Interv.* **6**, 148–165 (2004).

14. Kay, S. R., Wolkenfeld, F. M. & Murrill, L. M. Profiles of Aggression among Psychiatric Patients: I. Nature and Prevalence. *J. Nerv. Ment. Dis.* **176**, 539–546 (1988).
